# Identification of osteopontin-dependent signaling pathways in a mouse model of human breast cancer

**DOI:** 10.1186/1756-0500-2-119

**Published:** 2009-07-01

**Authors:** Zhiyong Mi, Hongtao Guo, Paul C Kuo

**Affiliations:** 1 Dept. of Surgery, Duke University Medical Center, Durham, NC, USA

## Abstract

**Background:**

Osteopontin (OPN) is a secreted phosphoprotein which functions as a cell attachment protein and cytokine that signals through two cell adhesion molecules, α_v_β_3_-integrin and CD44, to regulate cancer growth and metastasis. However, the signaling pathways associated with OPN have not been extensively characterized. In an in vivo xenograft model of MDA-MB-231 human breast cancer, we have previously demonstrated that ablation of circulating OPN with an RNA aptamer blocks interaction with its cell surface receptors to significantly inhibit adhesion, migration and invasion in vitro and local progression and distant metastases.

**Findings:**

In this study, we performed microarray analysis to compare the transcriptomes of primary tumor in the presence and absence of aptamer ablation of OPN. The results were corroborated with RT-PCR and Western blot analysis. Our results demonstrate that ablation of OPN cell surface receptor binding is associated with significant alteration in gene and protein expression critical in apoptosis, vascular endothelial growth factor (VEGF), platelet derived growth factor (PDGF), interleukin-10 (IL-10), granulocyte-macrophage colony stimulating factor (GM-CSF) and proliferation signaling pathways. Many of these proteins have not been previously associated with OPN.

**Conclusion:**

We conclude that secreted OPN regulates multiple signaling pathways critical for local tumor progression.

## Findings

Osteopontin (OPN), is a secreted phosphoprotein which signals through α_v_β_3_-integrin and CD44 to increase cellular migratory and invasive behavior, increase metastasis, promote colony formation and 3D growth ability, induce tumor-associated inflammatory cells, and induce expression of angiogenic factors. [1-3] Gain- and loss-of function assays have demonstrated a critical role for OPN in tumor metastatic function in colon, liver, and breast cancers. [3-5] However, OPN dependent signal transduction pathways have not been extensively studied in an in vivo setting.

Recently, we utilized an OPN directed RNA aptamer (OPN-R3) to inhibit in vivo and in vitro metastatic function of the MDA-MB231 human breast cancer cell line.[6] Our results indicated that RNA aptamer binding of OPN blocks interaction with its cell surface receptors to significantly inhibit adhesion, migration and invasion in vitro and local progression and distant metastases in an in vivo xenograft model. In the present study, we sought to build on our previous observations by determining alterations in the OPN-dependent signal transduction pathways that are mediated by RNA aptamer targeting of secreted OPN. Using specimens from our in vivo xenograft model of MDA-MB231 human breast cancer, we performed microarray analysis to compare the transcriptomes of primary tumor in the presence and absence of aptamer ablation of OPN. The microarray results were then corroborated with RT-PCR and Western blot analysis. Our data demonstrate that ablation of OPN cell surface receptor binding is associated with significant alteration in gene expression critical in apoptosis, vascular endothelial growth factor (VEGF), platelet derived growth factor (PDGF), interleukin-10 (IL-10), granulocyte-macrophage colony stimulating factor (GM-CSF) and proliferation signaling pathways. Many of these proteins have not been previously associated with OPN in breast cancer. We conclude that secreted OPN regulates multiple signal transduction pathways critical for local tumor progression in this in vivo model of human breast cancer.

## Methods

RNA aptamer and systematic evolution of ligands by exponential enrichment (SELEX): The SELEX selection procedure was utilized to isolate candidate OPN aptamers, as described previously.[7,8] After each round of SELEX, we performed a binding affinity assay to measure the aptamer pool's Kd value to ensure that the Kd values exhibited a decreasing trend. We applied SELEX by alternating the bait protein between human OPN and mouse OPN in order to achieve RNA aptamer targeting to common features of both proteins. The DNA sequence used for in vitro transcription was 5'-GGGGGAATTCTAATACGACTCACTATAGGGAGGACGATGCGG-N40-CAGACGACTCGCTGAGGATCCGAGA-3', where N40 represents the 40 nt RNA aptamer library sequence. The sequences for the aptamers are as follows:

OPN-R3: 5'-CGGCCACAGAAUGAAAAACCUCAUCGAUGUUGCAUAGUUG-3'

Mutant OPN-R3: 5'-CGGCCACAGAAU*GAAU*CAUCGAUGUUGCAUAGUUG-3'

where C denotes 2-OMe-dCTP and U denotes 2-OMe-dUTP, as appropriate. Commercially synthesized OPN-R3 aptamers contain 2'-OMe C, 2'-OMe U, A, and G and were used for in vivo studies.

### In vivo OPN-R3 activity

Animal handling and procedures were approved by the Duke University Animal Care and Use Committee. 6-week old female NOD scid mice were obtained from the Jackson Laboratory, Bar Harbor, ME. 1 × 10^6 ^MDA-MB-231-luciferase-expressing cells (a gift of Dr. Mark Dewhirst, Duke University, NC) were suspended in 50% Matrigel-Hanks balanced salt solution and implanted into the R4 or L4 positions of the mice mammary fat pad. Modified OPN-R3 and Mutant OPN-R3 (500 μg/kg) were injected into the mouse tail vein every two days following cell implantation. Mice were anesthetized with intraperitoneal ketamine (75 mg/kg) and xylazine (10 mg/kg) and subjected to bioluminescent imaging twice weekly to follow tumor progression. The volume of the primary tumors were quantified with caliper measurements in two dimensions and tumor volume (*V*) calculated using the following formula: *V *= (1/2) *S*^2 ^× *L *(*S*, the shortest dimension; *L*, the longest dimension). Primary tumor tissues were excised from OPN-R3 and Mutant OPN-R3 treated mice.

### cDNA microarray analysis

Total RNA was extracted from primary tumor using RNeasy mini kit (Qiagen, Valencia, CA). A total of nine animals were used (WT, n = 3; OPN-R3, n = 3; Mutant OPN-R3, n = 3). The cDNA synthesis, labeling, hybridization and scanning were performed by the Duke University Microarray Facility. The Human Operon v4.0 spotted array covering 35 k human genes was used; The complete description of the array is available at . Samples from each animal were arrayed separately. The raw microarray data can be accessed at . Microarray data were analyzed by the Partek Genomics Suite software (Partek, St. Louis, MO). Gene expression signal pathways were analyzed with the Ingenuity Pathways Analysis software (Ingenuity Systems, Redwood City, CA).

### Real-time PCR analysis

Real-time PCR was performed with the two-step reaction protocol using iQ SYBR Green detection kit (Bio-Rad Laboratories, Hercules, CA). First-strand cDNA were synthesized from 1-μg total RNA using the iScript Select cDNA synthesis kit (Bio-Rad Laboratories, Hercules, CA) at 48°C for 30 min. Glyceraldehyde 3-phosphate dehydrogenase (GAPDH) was used as the endogenous control. The primer sets were used for the quantitative PCR analysis are listed in Table 1. Real-time PCR parameters used were as follows: 95°C for 3 min; 95°C for 30 s, 55°C for 35 s for 40 cycles; 95°C for 1 min, and 55°C for 10 min. PCR was performed with iQ SYBR Green super mix, using the iCycler iQ Real-time PCR Detection System (Bio-Rad Laboratories, Hercules, CA). The 2-delta-delta Ct value was calculated following GAPDH normalization.

**Table 1:** Real-time PCR Primer Sets

GAPDH:	forward:5'-AGCCTCAAGATCATCAGCAATGCC-3', reverse: 5'-TGTGGTCATGAGTCCTTCCACGAT-3'.
Hypoxia inducible factor-1α (HIF-1α):	forward: 5'-GACTCAGCTATTCACCAAAG-3', reverse: 5'-AAAGATATGATTGTGTCTCC-3'

VEGF:	forward: 5'-ATCACGAAGTGGTGAAGTTC-3', reverse: 5'-AGGATGGCTTGAAGATGTAC-3'

PDGFα:	forward: 5'-GACACCAGCCTGAGAGCTCA-3', reverse: 5'-CCTGGTCTTGCAGACAGCGG-3'

SRC:	forward: 5'-GGCTGGAGGTCAAGCTGGGC-3', reverse: 5'-GGAAGGCCTCTGGAGACATC-3'

β-Catenin:	forward: 5'-GTCCATGGGTGGGACACAGC-3', reverse: 5'-CTGATAACAATTCGGTTGTG-3'

B-cell CLL/lymphoma-2 (BCL-2):	forward: 5'-GAGGTGATCCCCATGGCAGC-3', reverse: 5'-TGTCCCTGGGGTGATGTGGA-3'

Heme-oxygenase-1 (HO-1):	forward: 5'-TGTACCACATCTATGTGGCC-3', reverse: 5'-CCAGGTCCTGCTCCAGGGCA-3'

Signal Transducers and Activator of Transcription 3 (STAT3):	forward: 5'-CAGCAGATGCTGGAGCAGCA-3', reverse: 5'-CTTGAGGGTTTTATAGTTGA-3'

Oncostatin-M (OSM):	forward: 5'-GAAGCAGACAGATCTCATGC-3', reverse: 5'-CTCCCTGCAGTGCTCTCTCA-3'

Calmodulin-dependent protein kinase-2A (CAMK2A):	forward: 5'-GGAAGCCAAGGATCTGATCA-3', reverse: 5'-TGCATGCAGGATGCCACGGT-3'

B-cell translocation gene-3β (BTG3-β):	forward: 5'-AGGACAGGCCTACAGATGTA-3', reverse: 5'-GAGAGTGAGCTCCTTTGGCA-3'

Cluster of Differentiation 82 (CD82):	forward: 5'-AGAGCAGTTTCATCTCTGTC-3', reverse: 5'-GCAGCCCAGGAAGCCCATGA-3'

### Western blot analysis

Western blot analysis was performed as previously described.[9] The membranes were probed with the following primary antibodies for 1 h at room temperature: Src Ab (Cell Signaling, Beverly, MA), HIF-1 Ab, VEGF Ab, PDGF Ab, β-Catenin Ab, STAT3 Ab, CAMK2A Ab, OSM Ab, BTG3-b Ab, CD82 Ab, GAPDH Ab (Santa Cruz Biotechnology, Santa Cruz, CA), BCL-2-like 1 Ab, HO-1(Sigma-Aldrich, Atlanta, GA). Following exposure to horseradish peroxidase-conjugated secondary antibody, reactive proteins were visualized by means of the ECL kit (Amersham Biosciences, Piscataway, NJ).

### Statistical analysis

For microarray data analysis, the raw intensity values were exported to GeneSpring GX 10 software (Agilent Technologies, Santa Clara, CA). Normalization was achieved using a Z score transformation method on GeneSpring 10 software. This method ultimately produced a distribution of Z scores for all the genes in each array, and used the differences in the Z scores to calculate Z ratios to locate genes that were expressed differentially. The student t-test was applied for p-value calculation. Genes that had a Z ratio > 1.5 and a p-value < 0.005 were considered significant.

## Results and Discussion

We have previously demonstrated that the RNA aptamer, OPN-R3, blocks interaction with its cell surface receptors resulting in significantly decreased: 1) expression of MMP2 and uPA, 2) activation of CD44 and α_v_β_3 _dependent signal transduction pathways, 3) in vitro measures of adhesion, migration and invasion, and 4) in vivo local progression and distant metastases in a xenograft model.[6] Bioluminescence was significantly decreased in the OPN-R3 treated animals by over 4- and 12-fold at 20 and 30 days post-implantation, respectively. (p < 0.01 at 20 days and 30 days for OPN-R3 vs Mutant OPN-R3 and Vehicle) At day 20, tumor volume in the OPN-R3 aptamer treated group was > 18 fold smaller than that of Mutant OPN-R3 and Vehicle groups. (p < 0.01 vs. Mutant OPN-R3 and Vehicle) At day 30, OPN-R3 aptamer treated group tumor was 8-fold less than that of the Mutant OPN-R3 and the Vehicle groups. (p < 0.01 vs. Mutant OPN-R3 and Vehicle) These data indicate that OPN-R3 aptamer can significantly decrease local tumor growth of MDA-MB231 cells in this xenograft model and were previously reported.

**Figure 1:**
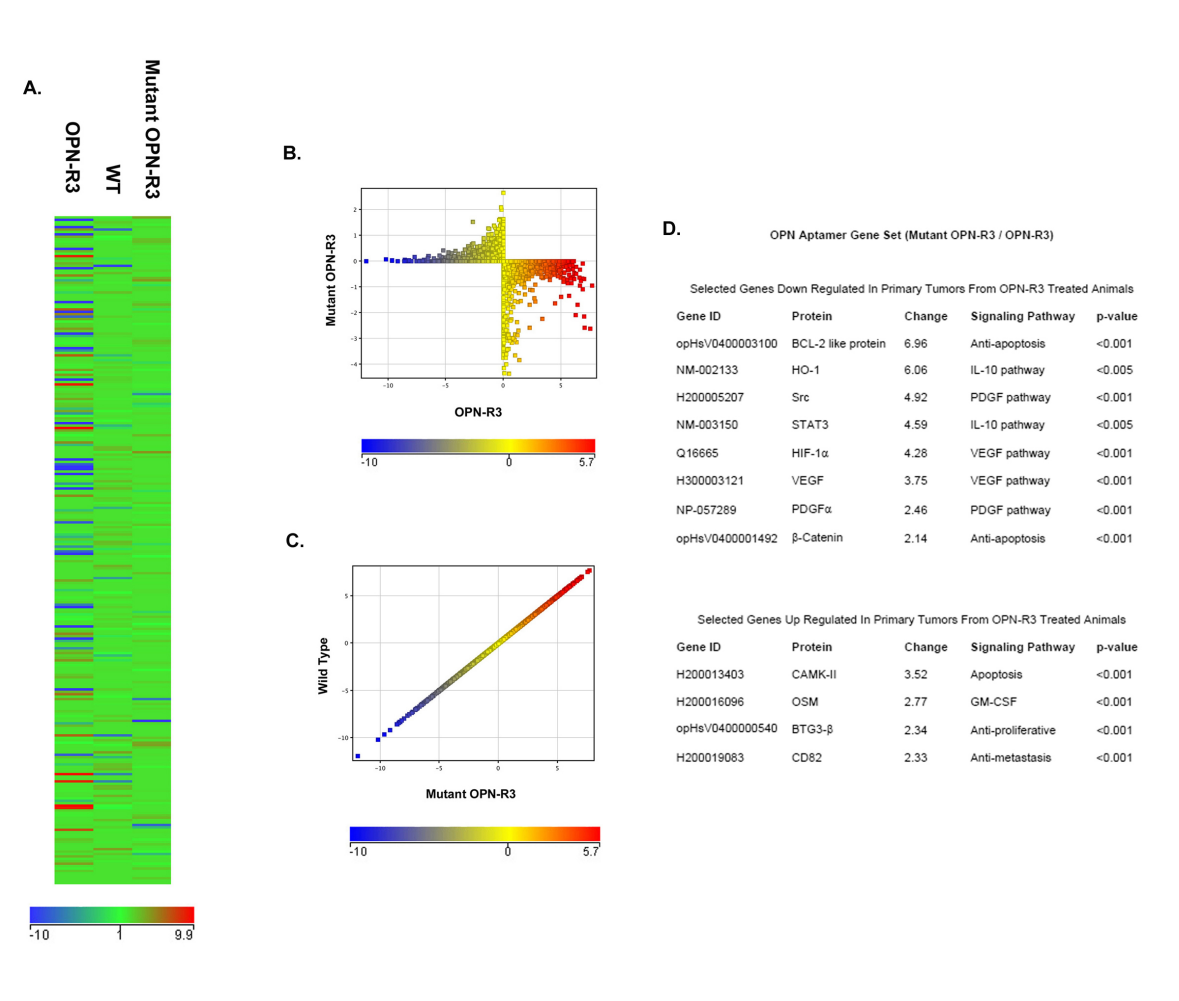
**Gene expression profiling**. A. Microarray heat map analysis of mouse primary tumors treated by OPN-R3(left); wild type non-treatment (middle) and Mutant OPN-R3 aptamer. The panel shows gene expression fold change compared with the mean normalized value of controls (wild type non-treatment and mutant OPN-R3 aptamer treatment). The intensity of the node color indicates the degree of increase (red) or decrease (blue) gene expression. B, C. Scatter plots showing differentially expressed genes between mutant OPN-R3 aptamer treatment and OPN-R3 aptamer treatment (B) and between wild type non-treatment and mutant OPN-R3 treatment control(C). D. List of the dysregulated genes associated with down regulated and up regulated canonical signal transduction pathways.

**Figure 2:**
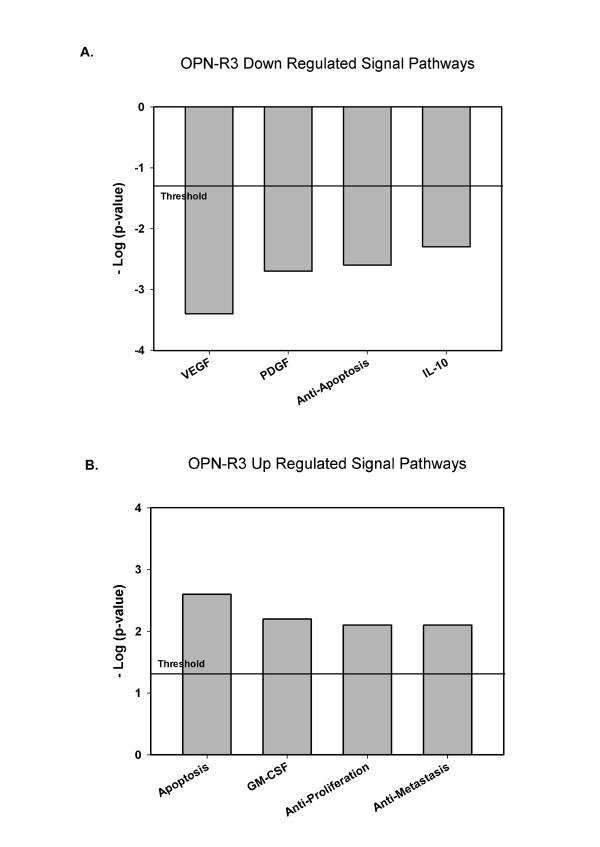
**Ingenuity Pathways Analysis**. The bar graphs indicate down regulated and up regulated canonical pathways associated with OPN-R3 aptamer treatment. A. Four down regulated canonical biochemical and molecular biology pathways with significant (p < 0.05, Fisher's exact test) correlation in comparison to the wild type non-treatment and Mutant OPN-R3 aptamer treatment controls. B. Four up regulated canonical biochemical and molecular biology pathways with significant (p < 0.05, Fisher's exact test) correlation in comparison to the wild type non-treatment and Mutant OPN-R3 aptamer treatment controls.

Based upon our previously published findings (as detailed above), we next sought to identify the genes whose expression is regulated by OPN in this model, we extracted RNA from primary tumor from untreated (WT) animals and those treated with OPN-R3 and Mutant OPN-R3. The reference set was defined to be the mean of the WT and Mutant OPN-R3 groups. The heat map of the three groups and the scatter plots of WT versus Mutant OPN-R3 and OPN-R3 versus Mutant OPN-R3 are displayed in Figure 1. The scatter plots indicate that significant differences in gene expression are present between the OPN-R3 and Mutant OPN-R3 groups, while the WT and Mutant OPN-R3 groups are not significantly different. The top eight genes down-regulated by >2-fold and the top four genes up-regulated by >2-fold primary tumors from OPN-R3 treated animals are listed in Figure 1D. Genes were then assigned to biological pathways using Ingenuity Pathway software. (Figure 2) The threshold value of -log (p-value) was set at 1.31, corresponding to a p-value of 0.05. This software suggests that OPN-R3 was associated with down regulation of IL-10, VEGF, PDGF and anti-apoptosis signaling with concomitant up regulation of apoptosis, GM-CSF, anti-proliferative and anti-metastasis signaling pathways.

**Figure 3:**
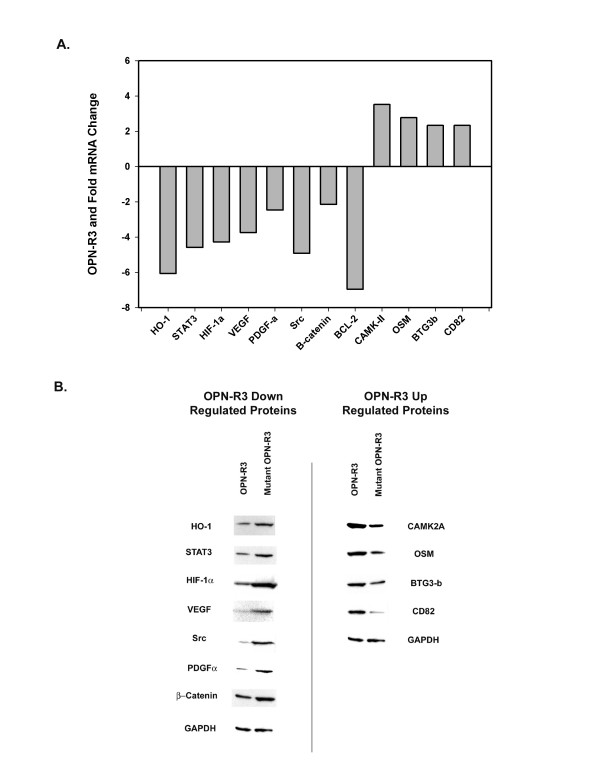
**Verification of Microarray Results**. A. Histogram of mRNA changes in MDA-MB231 primary tumor from animals treated with OPN-R3 and/or Mutant OPN-R3. Real-time PCR was performed and the 2-delta-delta Ct value was calculated following GAPDH normalization. Fold induction was determined relative to cells treated with Mutant OPN-R3. A total of six animals were analyzed (OPN-R3, n = 3; Mutant OPN-R3, n = 3). Data are representative of three experiments. B. Western blots of differentially expressed proteins in MDA-MB231 primary tumor from animals treated with OPN-R3 and/or Mutant OPN-R3. Blots are representative of three experiments.

The microarray data suggest that OPN-R3 treatment was associated with decreased expression of genes involved in IL-10 (HO-1, STAT3), VEGF (HIF-1α, VEGF), PDGF (PDGF-α, Src) and anti-apoptosis (β-catenin, BCL-2 like protein) signaling and increased expression of genes involving apoptosis (CAMK2A), GM-CSF (OSM), anti-proliferative (BTG3-b) and anti-metastasis (CD82) signaling. Real-time RT-PCR and Western blot analysis were used to verify altered expression of the identified genes and proteins in OPN-R3 and Mutant OPN-R3 groups. RT-PCR corroborated the microarray results. (Figure 3A) Similarly, expression of the corresponding proteins were also altered in a fashion predicted by the microarray results. (Figure 3B) These results demonstrate that blockade of OPN binding through RNA aptamer targeting decreases expression of key proteins involved in the IL-10, VEGF, PDGF and anti-apoptosis pathways with simultaneous increases in apoptosis, GM-CSF, anti-proliferative and anti-metastasis signaling proteins.

OPN was initially characterized in 1979 as a phosphoprotein secreted by transformed, malignant epithelial cells.[10,11] It is a member of the small integrin-binding ligand N-linked glycoprotein (SIBLING) family of proteins which include bone sialoprotein (BSP), dentin matrix protein 1 (DMP1), dentin sialoprotein (DSPP), and matrix extracellular phosphoglycoprotein (MEPE).[1,12,13] The molecular structure of OPN is rich in aspartate and sialic-acid residues and contains unique functional domains which mediate critical cell-matrix and cell-cell signaling through the α_v_β_3 _integrin and CD44 receptors in a variety of normal and pathologic processes. [14-19] As a secreted protein, OPN represents an ideal target for OPN-R3 aptamer mediated inhibition. In this study examining the transcriptomes of primary tumors from OPN-R3 treated animals, we show that ablation of OPN cell surface binding decreases expression of key proteins involved in the IL-10, VEGF, PDGF and anti-apoptosis pathways with simultaneous increases in apoptosis, GM-CSF, anti-proliferative and anti-metastasis signaling proteins. These results indicate the wide ranging effects of OPN in regulating critical pathways required for local tumor growth and metastasis.

Review of the specific genes identified in this study indicates that, in certain instances, associations with OPN and cancer have been identified in the past. For example, Src is a well recognized member of the signal transduction pathway associated with OPN binding to CD44.[6] However, the relationship between OPN and other genes is not as well characterized. Recently, in a variety of in vitro and in vivo assay using MDA-MB-231 cells, Chakraborty et al. found that OPN plays a key role in VEGF expression and VEGF-stimulated neovascularization.[20,21] In advanced head-and-neck cancer, a poor prognosis correlated with parallel increases in both OPN and HIF-1α. [22,23] Siddiqui and coworkers found that STAT3 was increased while OPN was decreased in response to green tea polyphenol in a mouse model of prostate cancer.[24] In a nude mouse model of hepatocellular cancer, Zhao and colleagues demonstrated that OPN silencing using shRNA inhibited BCL-2 protein expression.[5] The relationship between β-catenin and OPN has been extensively documented.[1,25-27] T cell factor 4 (Tcf-4) interacts with beta-catenin in the Wnt signaling pathway and coactivates downstream target genes in the breast. In a rat model for breast cancer, it has been shown that metastasis-inducing DNA sequesters the endogenous inhibitory Tcf-4 and thereby promotes transcription of OPN.[1,27] However, at present, it is unclear why ablation of extracellular OPN would result in decreased β-catenin mRNA and protein expression. Many of the studies merely document associations without demonstration of causality or delineation of mechanism. HO-1, OSM, CAMK-2, CD82, BTG3-β and PDGF have not been previously linked with OPN in the setting of cancer.

While these results require confirmation in other cell types and human tissues, our study indicates that OPN regulates the local growth and metastasis through a complex multi-faceted signaling network. Characterization of these pathways using our functional model identifies potential targets for future therapies directed agaqinst OPN signaling in breast cancer.

## Competing interests

The authors declare that they have no competing interests.

## Authors' contributions

ZM and HG made substantial contributions to conception and design, acquisition of data, analysis and interpretation of data. PK made substantial contributions to conception and design, analysis and interpretation of data, and wrote the manuscript. All authors read and approved the final manuscript.
